# Lessons learned from the conduct of a multisite cluster randomized practical trial of decision aids in rural and suburban primary care practices

**DOI:** 10.1186/1745-6215-14-267

**Published:** 2013-08-21

**Authors:** Kari L Ruud, Annie LeBlanc, Rebecca J Mullan, Laurie J Pencille, Kristina Tiedje, Megan E Branda, Holly K Van Houten, Sara R Heim, Margary Kurland, Nilay D Shah, Barbara P Yawn, Victor M Montori

**Affiliations:** 1 Knowledge and Evaluation Research Unit, Mayo Clinic, Rochester, MN, USA; 2 Department of Health Sciences Research, Division of Health Care Policy and Research, Mayo Clinic, Rochester, MN, USA; 3 Center for the Science of Healthcare Delivery, Mayo Clinic, Rochester, MN, USA; 4 University of Minnesota Medical School, Minneapolis, MN, USA; 5 Department of Medicine, Division of Endocrinology, Mayo Clinic, Rochester, MN, USA; 6 Department of Anthropology and Sociology, Université Lumière Lyon 2, Lyon, France; 7 Department of Research, Olmsted Medical Center, Rochester, MN, USA; 8 Department of Family and Community Health, University of Minnesota, Minnesota, MN, USA

**Keywords:** Practical trials, Trial methodology, Recruitment and enrollment

## Abstract

**Background:**

The decision aids for diabetes (DAD) trial explored the feasibility of testing the effectiveness of decision aids (DAs) about coronary prevention and diabetes medications in community-based primary care practices, including rural clinics that care for patients with type 2 diabetes.

**Methods:**

As originally designed, we invited clinicians in eight practices to participate in the trial, reviewed the patient panel of clinicians who accepted our invitation for potentially eligible patients, and contacted these patients by phone, enrolling those who accepted our invitation. As enrollment failed to meet targets, we recruited four new practices. After discussing the study with the clinicians and receiving their support, we reviewed all clinic panels for potentially eligible patients. Clinicians were approached to confirm participation and patient eligibility, and patients were approached before their visit to provide written informed consent. This in-clinic approach required study coordinators to travel and stay longer at the clinics as well as to screen more patient records for eligibility. The in-clinic approach was associated with better recruitment rates, lower patient retention and outcome completion rates, and a better intervention effect.

**Results:**

We drew four lessons: 1) difficulties identifying potentially eligible patients threaten the viability of practical trials of DAs; 2) to improve the recruitment yield, recruit clinicians and patients for the study at the clinic, just before their visit; 3) approaches that improve recruitment may be associated with reduced retention and survey response; and 4) procedures that involve working closely with the practice may improve recruitment and may also affect the quality of the implementation of the interventions.

**Conclusion:**

Success in practice-based trials in usual primary care including rural clinics may require the smallest possible research footprint on the practice while implementing a streamlined protocol favoring in-clinic, in-person interactions with clinicians and patients.

**Trial registration:**

ClinicalTrials.gov NCT01029288

## Background

Shared decision making (SDM) and patient-centered care are now widely recognized as desirable components of a high quality healthcare system. A systematic review of 86 randomized trials confirms the ability of SDM tools or decision aids (DAs) to promote patient involvement in decision making [1]. This has motivated policies and legislation promoting patient participation in decision making and the use of DAs in practice, including the Patient Protection and Affordable Care Act (2010), the so-called US healthcare reform law [2]. How best to implement these effective tools in clinical practice remains uncertain [3]. Moreover, DA trials have been conducted primarily in university settings and often outside of clinical contexts, distinct from primary care practices in general and of rural practices in particular [4].

The conduct of randomized controlled trials (RCTs) in usual practice settings can present challenges, particularly concerning patient recruitment. Many clinical trials either fail to meet recruitment goals within the intended timeframe or do not meet recruitment goals at all [5-9]. This can pose ethical and financial challenges such as limiting the statistical strength of study results, increasing study costs, and exposing study participants to risk despite a diminished ability to answer the study question with sufficient precision [5,8]. Over 50% of studies have been reported to require extensions due to insufficient recruitment [10]. While a Cochrane systematic review identified telephone reminders to non-responders, opt-out procedures, and financial incentives as potential methods for increasing enrollment [5], there is limited evidence regarding generalizable strategies to improve recruitment [9,11]. Furthermore, RCTs of DA tools in rural practices face particular challenges: clinical sites can be separated by vast distances, rural clinicians may lack familiarity with SDM and DA tools and with participating in clinical care research, and patients are less likely to be insured (have unfettered access to the practice) and have experience with participation in research activities [4].

To evaluate the effectiveness of our diabetes-focused DAs in primary care clinics in rural and suburban southeastern Minnesota, USA, we undertook a multicenter, pragmatic RCT. This paper describes some lessons we have drawn from its conduct.

## Methods

Detailed accounts of the study protocol are published elsewhere and are briefly reported below [12].

### Study design and setting

We conducted a cluster randomized, practical, multicenter trial, enrolling rural and suburban primary care practices in the Midwestern United States. Participating primary care practices were matched by size and randomized to the intervention or usual care (UC) arm by an external statistician. Practices were randomized to one of three DAs (available at http://shareddecisions.mayoclinic.org): Statin Choice or Aspirin Choice (that is, cardiovascular medication choices) or Diabetes Medication Choice, serving as control for the practices allocated to the other DA. Institutional Review Boards (IRBs) at participating sites approved all study procedures. The study is registered at ClinicalTrials.gov (Identifier: NCT01029288).

### Participants

Primary care practices were considered for enrollment if they were located within rural and suburban areas, and within a 50-mile radius from the research team’s primary location in Rochester, MN, USA. Clinicians (that is, physicians, nurse practitioners, physician assistants) from participating practices were eligible if they provided care for adults with type 2 diabetes. English speaking patients (≥18 years old) were considered eligible if they had type 2 diabetes, were considered by their clinician to have inadequate glucose control and be at the top of the dose of medication related to the management of their cholesterol or diabetes, and had no major barriers to providing written informed consent. In other words, we sought patients in whom it was necessary and possible to have a discussion about medications. Additional criteria were required according to each of the arms, and a flowchart was developed to facilitate the process of identification of eligible patients (available at http://www.ncbi.nlm.nih.gov/pmc/articles/PMC3468357/figure/F2/) [12].

### Identification and selection for recruitment

Primary care practices were contacted at which investigators had a direct link or knew potential clinical champions to seek interest in participating in the trial. Primary care clinicians were approached before the start of the study during an initial meeting between practice staff and research team members. Clinicians were offered to participate in the study and signed written informed consent documents during this meeting. Clinicians who chose not to participate or were absent from this meeting were not approached again. No financial incentive was offered to clinicians or practices.

Study coordinators sought eligible patients with upcoming appointments with participating clinicians using the practice’s diabetes registry and medical records. They contacted eligible patients by telephone 1 week before their scheduled appointment. After three unsuccessful phone calls, it was assumed these patients declined participation. To enroll patients who had agreed to participate, study coordinators traveled to practice sites to obtain written consent, and proceed with study enrollment and assessment. Mailed follow-up surveys and pharmacy record tracking were handled by a central study coordinator. A two-dollar bill (US$2) was included with each follow-up survey sent to participating patients.

### Trial strategies

The study team aimed to keep the disruption of workflow and burden on clinicians and staff to a minimum. Study coordinators, who were located in a central office away from the study sites, were responsible for all aspects of patient recruitment and follow-up. Onsite visits by study coordinators were limited to when eligible patients had previously agreed to meet. Clinicians were notified by email when one of their patients was eligible for the study and that a study coordinator would be present at the upcoming appointment. Further contact was limited, and patient and clinician surveys were kept to a minimal number and length.

### Intervention and usual care (UC)

The intervention consisted of the use of a DA (Diabetes Medication or Statin or Aspirin Choice) by patients and their primary care clinicians during a regular clinical encounter [12-15]. Clinicians were briefly trained by the investigators during an initial meeting, and study team members remained available for one-on-one demonstration when needed. Online access to a brief video clip and storyboard demonstrating the use of DAs was also provided. Clinicians were handed the DA just before a clinical encounter with participating patients. Participating clinicians and patients in the UC arm discussed medication regimens as usual. All DAs and related material can be found online at http://shareddecisions.mayoclinic.org/.

### Data collection and outcome measures

Surveys were administered to both clinicians and patients immediately following the visit, and follow-up surveys were sent to patients by mail 3 and 6 months following the visit. Information about diabetes-related care was extracted from the medical record. With clinician and patient approval, we videotaped encounters using a small, easy-to-use video camera. Primary outcome measures were: the patient’s level of comfort with the decision made (measured using the decisional conflict scale (DCS) [16]), the extent to which clinicians involved patients in the decision making process (using the observing patient involvement (OPTION) scale [17]), and patient’s knowledge of risks and benefits relevant to the decision they faced. Other outcomes included patient satisfaction, quality of life, degree of metabolic control, adherence to medication, and clinician’s satisfaction [12]. We reviewed video recordings of encounters using checklists specific to each DA to assess the fidelity with which clinicians were able to use the DA as intended or to assess the extent to which clinicians demonstrated similar behaviors when providing UC suggesting potential contamination across arms.

### Sample size and analysis

To detect a meaningful difference in our primary outcome of interest (decisional comfort), we needed to enroll eight practices and 240 patients. Before study start, we ascertained the eligible patient population from analyses of practice databases and estimated that a 9-month period would be needed to achieve our recruitment goal. Details of the analysis plan and results are reported elsewhere [12,13].

### Identification of lessons learned

The project manager and the lead study coordinator kept detailed notes during the conduct of the trial. Minutes were maintained for the periodic research meetings in which investigators and study personnel reviewed study progress and solved problems as they arose. Comments and observations relayed to the team in person or through electronic communication by study participants were also saved. All material was maintained and issues tracked. Some issues were identified as impactful enough that they led to changes in the conduct of this trial and/or affected the design and execution of the team’s other trials of DAs in primary care; these issues are presented here as lessons learned.

## Results

### Lesson 1: difficulties identifying potentially eligible patients threaten the viability of practical trials of decision aids (DAs)

We designed our study with the intention to optimize recruitment and minimize bias, while considering the time, effort, and commitment that study participation demands of practices and clinicians. Each practice was allocated to a specific DA within the intervention arm, while serving as the UC arm for the remaining DAs. This design allowed the opportunity for each practice to take part in the intervention and eliminated the need for any practice to serve only as an UC arm site. Moreover, this design ensured that DAs were implemented in all participating practices, preventing randomization from fortuitously allocating the implementation of DAs preferentially to more flexible and organized practices. However, we had not anticipated how arduously our study coordinators would have to work to identify eligible patients. They had to review the medical record of each potentially eligible patient, ascertaining the diagnosis of diabetes, HbA1c value, diabetes medication history, use of aspirin, and use of statins. This time-intensive process took about 25 minutes per candidate. In particular, determining whether patients were taking aspirin required record review because aspirin is not a reimbursable prescription and information about its use is therefore not included in the e-prescription section of the medical record. Coordinators found that most candidates were already taking aspirin. In consideration of the significant amount of time required to review aspirin information and the limited number of available patients not taking aspirin, the study team decided to remove the Aspirin Choice DA approximately 1 month into the study, at which point only two patients had been assigned to this DA. This modification streamlined the process for identifying eligible patients and considerably reduced screening time required of study coordinators.

### Lesson 2: to improve the recruitment yield, recruit clinicians and patients for the study at the clinic, just before their visit

**Figure 1:**
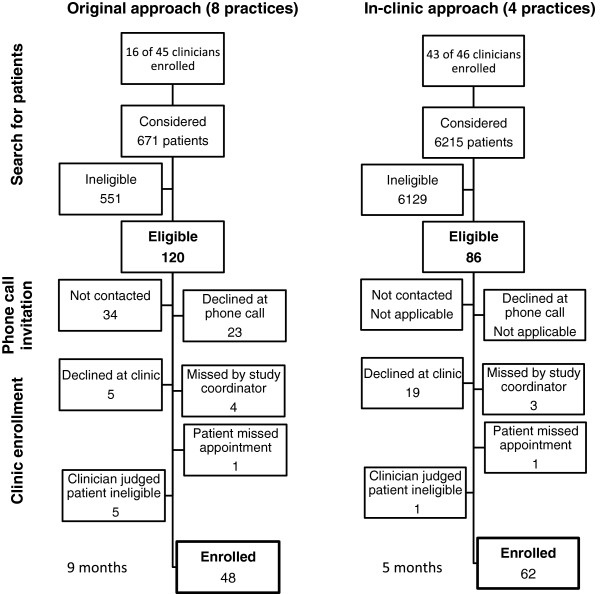
**Enrollment flow associated with the recruitment approaches used during the DAD trial.** Left, original design; right, in-clinic modified approach. DAD, decision aids for diabetes.

We contacted known rural and suburban primary care practices and presented the study to practice members: eight practices agreed and three declined study participation. In keeping with our objective to be minimally disruptive, clinicians at participating practices were approached individually only once to invite their participation. Figure 1 (left) shows the study performance during the 9 months of recruitment leading to the identification of 121 eligible patients and the successful enrollment of 48 patients.

**Figure 2:**
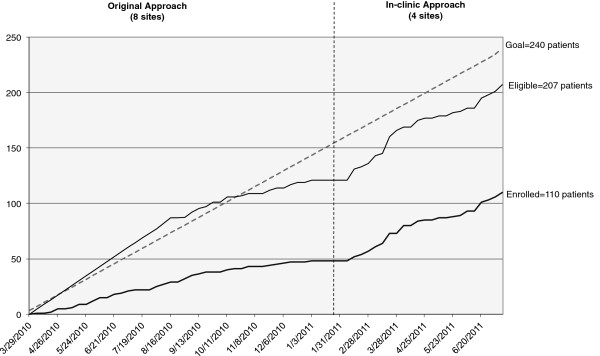
**Eligibility and enrollment of patients over time in the DAD trial.** Left, original design; right, in-clinic modified approach. DAD, decision aids for diabetes.

Over time, we saw plateauing of the eligible patient population (Figure 2) and realized that trial enrollment targets could not be met, leading to a revision of clinician and patient recruitment protocols. The study team decided not to re-contact clinicians who had already declined participation. Modifying patient eligibility criteria was considered, but expansion of the eligibility criteria would threaten the pertinence of the intervention. Thus, it was determined that further attempts at patient enrollment would not be an effective use of resources, and recruitment was discontinued at the initial eight sites.

Four new suburban practices were approached using a different method (Figure 1, right). Clinicians were offered the opportunity to give written informed consent at the initial meeting with the study team, as in the original recruitment procedure. However, in scanning registries and records for potentially eligible patients, study coordinators considered all practice patients with diabetes with upcoming appointments, only avoiding patients from clinicians who had declined participation at the initial visit. If an eligible patient was identified within the panel of a clinician who had not yet given consent for participation, the study coordinators approached the clinician at their next encounter. These clinicians were offered the options of consenting to participation, delaying their participation in the study if the timing was not appropriate (study coordinator could approach them again for future eligible patients), or declining participation definitively. In these practices, we discontinued phone interviews with candidate patients, instead approaching them immediately as they appeared for their scheduled appointment at the clinic. During the interview in the clinic examination room – unhurried, quiet, private, and face-to-face – study coordinators reviewed details of the study and obtained written informed consent. Table 1 offers a summary of the differences between the two recruitment approaches.

**Table 1:** Comparison of recruitment methods

	**Original approach (n = 8 sites)**	**In-clinic approach (n = 4 sites)**
**Clinician recruitment**	Primary care clinicians approached and consented before start of study	Primary care clinicians approached and consented before start of study and/or at first eligible study visit
**Patient recruitment**	Eligible patients recruited by study coordinator by telephone 1 week before appointment	Eligible patients recruited by study coordinators in person at appointment
**Screening procedure**	Screening for eligible patients from listings of upcoming diabetes appointments with participating clinicians	Screening for eligible patients from registry of all diabetic patients at site

The in-clinic approach in the four new sites continued for 5 months. Figure 1 (right) shows the study performance during the 5 months of recruitment leading to the identification of 86 eligible patients and the successful recruitment of 62 of them. Thus, the in-clinic approach was superior to the original approach in proportion of eligible patients enrolled: 62 of 86 (72%) compared with 48 of 121 (40%) with the original approach. This was due to a higher proportion of eligible patients who could not be reached using the telephone discussion method and to fewer patients who declined to participate when invited in person (Figure 1).

### Lesson 3: approaches that improve recruitment may be associated with reduced retention and survey response

Given the slow and laborious enrollment of patients into the study, participant retention was deemed essential since loss to follow-up would further impede results [18]. Survey participation rates for clinicians and patients were compared between the two approaches (Table 2). Significant differences were noted in survey return rates for 3-month follow-up (84% versus 66%, *P* = 0.03) and 6-month follow-up (71% versus 47%, *P* = 0.01), with the original approach yielding higher return rates. Clinician post-visit survey return rates also differed (100% versus 86%, *P* <0.01), with higher return rates at the initial practices. Thus, improvements in recruitment may have come at the expense of the intention-to-treat principle.

**Table 2:** Outcome completion rate associated with the recruitment approaches used during the DAD trial

	**Original approach**	**In-clinic approach**	***P*value**
**Patients enrolled, n (% of eligible set)**	48 (40)	62 (72)	<0.0001
**Patient survey**			
Patient post-visit survey return rate, n (%)^a^	45 (100)	54 (93)	0.07
Patient 3-month survey return rate, n (%)	38 (84)	38 (66)	0.03
Patient 6-month survey return rate, n (%)	32 (71)	27 (47)	0.01
**Clinician survey**			
Clinician survey return rate, n (%)	45 (100)	50 (86)	0.01
**Qualitative interview**			
Patients that agreed to qualitative interview, n (%)	18 (40)	22 (38)	0.96
**Encounter video recording**			
Patients that agreed to have the encounter video-recorded, n (%)	19 (42)	23 (40)	0.79

^a^Based on 45 and 58 patients used in analysis, respectively. *DAD* decision aids for diabetes.

### Lesson 4: procedures that involve working closely with the practice may improve recruitment and may also affect the quality of the implementation of the interventions

The post-encounter surveys asked patients and clinicians about their encounter experiences, specifically whether a discussion regarding medication occurred. No difference was found in the UC arms between the recruitment approaches, but large differences were evident in the outcomes between sites using different procedures. At the original sites, only 56% of patients in the DA arm reported a discussion, while 88% of patients reported a discussion at the in-clinic recruitment sites (*P* = 0.02; Table 3). Although a similar proportion of clinicians recruited through the two approaches reported the DA was easy to use (79% versus 85%, *P* = 0.7) and implemented their allocated intervention with similar fidelity, a much larger proportion found it easier to integrate the DA into their practice in the in-clinic group (46% versus 97%, *P* <0.01). Clinicians in the first eight sites in both control and intervention arms achieved significantly less patient involvement as observed on video-recorded encounters than clinicians recruited through the in-clinic approach (mean OPTION score 16.3 versus 34.8 in the control arms; 38.7 versus 62.8 in the DA arms, respectively; Table 3).

**Table 3:** Implementation and integration of the decision aid (DA) compared to usual care (UC) in practices participating in the DAD trial

	**Original approach**	**In-clinic approach**	***P*value**
**Videos/patients used in analysis, n (%)**	18/45 (40)	23/58 (40)	0.89
**Fidelity score, mean (SD)**			
UC	11.4 (22.7)	22.3 (16.6)	0.97
DA	65.2 (28.5)	66.3 (26.2)	0.24
**OPTION score, mean (SD)**			
UC	16.3 (13.9)	34.8 (28.9)	0.10
DA	38.7 (21.1)	62.8 (8.5)	0.003
**Patient-reported outcomes, n/N (%)**			
Had a discussion about medication			
UC	11/27 (41)	10/20 (50)	0.57
DA	10/18 (56)	30/34 (88)	0.02
**Clinician-reported outcomes, n/N (%)**			
Ease of use of DA	11/14 (79)	28/33 (85)	0.68
Easy to integrate into the practice	6/13 (46)	32/33 (97)	<0.01

*DA* decision aid, *DAD* decision aids for diabetes, *OPTION* observing patient involvement; *UC* usual care.

## Discussion

### Our findings

For this RCT in which we implemented an intervention into primary care practices, two methods were employed for recruitment that yielded different results. Both approaches minimized burden to participating sites and clinicians by placing the responsibility of recruitment on the study team, which has been shown to facilitate recruitment rates in a systematic review [19]. The original approach to clinician and patient recruitment was implemented at eight primary care sites. The rationale was to enroll committed clinicians in order to minimize disruption to the practice of less-interested colleagues. This process also made study coordinators more efficient by limiting the search for eligible patients to the panel of participating clinicians and limiting travel to meet with patients who had already expressed interest in participating. With the processes used at the subsequent four sites, the focus was an in-clinic approach for clinician enrollment, a more laborious patient identification process screening all the practice patients, and a more constant presence of study coordinators at the sites. These approaches differed in efficiency, recruitment, retention, implementation of the intervention, and outcomes. There are some potentially plausible explanations for our findings that could represent lessons learned for us and other investigators conducting clustered trials of practice interventions.

Before turning our attention to those lessons, we must remember that any comparisons drawn here are those of an observational study of the implementation of a pilot randomized trial. Given that the practices were not randomly allocated to the recruitment approaches, we cannot draw causal inferences linking the recruitment approaches with the outcomes observed. The validity of hypotheses linking differences in the clinics in which we used different recruitment methods – differences in location, interest in the study, or practice style – with recruitment outcomes weaken our inferences attributing these differences in recruitment to the different approaches used.

### Phone contact to initiate enrollment

While it may be efficient, calling patients at home to invite them to participate in the study may have hindered recruitment: almost two in every five patients declined with this method. Furthermore, we also missed enrollment opportunities because we considered patients had declined participation if they could not be reached by telephone or return our call after three attempts. If this method must be used – and there is a strong support for patients to reflect on the pros and cons of participation away from the anxieties of a clinic appointment – perhaps an introduction to the study before the telephone call, such as by an informational mail-out flyer, may have increased patients’ willingness to participate [19,20].

### Efficient use of study coordinators

Limiting patient search to the panels of participating clinicians certainly improved the efficiency of study coordinators, as did contacting potentially eligible patients in advance by telephone and limiting travel to consent patients who had already agreed to participate. Although this approach resulted in lower enrollment and higher decline rates than the more time-intensive in-clinic recruitment method, it yielded higher retention rates and significantly higher survey response rates. This may indicate a more thought-out decision to participate and a higher level of patient engagement when more time is afforded for building rapport with potential participants, as has been demonstrated in other studies [21,22]. Since we did not try the in-clinic approach in these clinics, we cannot know whether it would have had the same consequences it did on the clinics we approached later, or if clinicians and their patients would have found it acceptable.

Before study implementation, we reviewed each site’s diabetes registry and found sufficient patients in participating practices to meet our accrual goal. However, we found fewer eligible patients than needed to meet our goal (Figure 1), primarily because some clinicians at these practices declined to participate, excluding their patients from this study. This contributed to limited accrual at a number of sites, with less than three patients enrolled at sites where only one or two clinicians agreed to participate. Also, to avoid disturbances, we did not return to the site to try to recruit clinicians who had decided not to participate after the initial onsite training session. Perhaps the ongoing interaction between study coordinators and clinicians recruited through the in-clinic approach reinforced clinicians’ understanding of the work, thus resulting in better integration of the DA and of the trial procedures into the practice, higher levels of patient engagement in decision making, and more recognition by patients that a discussion regarding medication took place [23].

We believe that the ideal approach to successfully conducting a practice-based trial may depend on the nature of patients, clinicians, and practices of the interventions tested, and of the goals of the trial; that is, where it lies on the continuum from explanatory (testing efficacy under tightly controlled conditions to advance science) to practical (testing effectiveness under usual conditions to support the evidence needs of decision makers) [24]. We think our findings support the notion that strategies to identify and address barriers to clinical trial recruitment and retention while optimizing resource utilization involve difficult tradeoffs and may have unintended consequences, including affecting the effectiveness of the intervention, particularly when participant motivation matters.

## Conclusions

Successful practice-based trials in rural clinics require methodological decisions associated with tough tradeoffs. As originally designed, poor clinician recruitment severely restricted access to eligible patients, limited contact of researchers with the practice may have reduced clinician motivation, and patient recruitment practices may have recruited more ‘high quality’ patients, but too few to meet recruitment targets.

Although it takes more time, more work, and more travel for study coordinators, onsite recruitment offers important advantages. Onsite recruitment increased the face time investigators had with practice personnel, increasing familiarity and collaboration. A streamlined study design and recruitment protocol favoring in-person interactions with clinicians and patients has the potential to promote better enrollment and integration of the trial procedures into the fabric of the practices. Ultimately, the value of this approach is subject to judgments that we expect the insights presented here will help inform.

## Abbreviations

DA: Decision aid; DAD: Decision aids for diabetes; DCS: Decisional conflict scale; IRB: Institutional Review Board; OPTION: Observing patient involvement; RCT: Randomized controlled trial; SDM: Shared decision making; UC: Usual care.

## Competing interests

The authors declare that they have no competing interests.

## Authors’ contributions

KLR managed the study and drafted the manuscript. AL managed the conduct of the study and participated in drafting the manuscript. RJM participated in the design and conduct of the study, and in drafting the manuscript. LJP managed the conduct of the study, enrolled patients, and participated in drafting the manuscript. KT participated in the design of the study and drafting the manuscript. MEB participated in the design of the study, performed the statistical analysis, and participated in drafting the manuscript. HKV performed the statistical analysis and participated in drafting the manuscript. SH participated in the conduct of the study and enrolled patients. MK managed the conduct of the study. NDS participated in the design of the study. BY conceived the study, and participated in the design of the study and in drafting the manuscript. VMM conceived the study, and participated in the design of the study and in drafting the manuscript. All authors read and approved the final manuscript.
